# Concept mapping enhances learning of biochemistry

**DOI:** 10.3402/meo.v18i0.20157

**Published:** 2013-03-05

**Authors:** Krishna M. Surapaneni, Ara Tekian

**Affiliations:** 1 Department of Biochemistry, Faculty of Medicine, Saveetha Medical College and Hospital, Saveetha University, Chennai, India; 2 Department of Medical Education, College of Medicine, University of Illinois at Chicago, IL, USA

**Keywords:** didactic lectures, concept mapping, clinical cases, clinical biochemistry, undergraduate medical curriculum, small group teaching, medical education

## Abstract

**Background:**

Teaching basic science courses is challenging in undergraduate medical education because of the ubiquitous use of didactic lectures and reward for recall of factual information during examinations. The purpose of this study is to introduce concept maps with clinical cases (the innovative program) to improve learning of biochemistry course content.

**Methods:**

Participants were first year medical students (*n*=150) from Saveetha Medical College and Hospital (India); they were randomly divided into two groups of 75, one group attending the traditional program, the other the innovative program. Student performance was measured using three written knowledge tests (each with a maximum score of 20). The students also evaluated the relevance of the learning process using a 12-item questionnaire.

**Results:**

Students in the innovative program using concept mapping outperformed those in the traditional didactic program (means of 7.13–8.28 vs. 12.33–13.93, *p*<0.001). The students gave high positive ratings for the innovative course (93–100% agreement).

**Conclusion:**

The new concept-mapping program resulted in higher academic performance compared to the traditional course and was perceived favorably by the students. They especially valued the use of concept mapping as learning tools to foster the relevance of biochemistry to clinical practice, and to enhance their reasoning and learning skills, as well as their deeper understanding for biochemistry.

## Introduction

Anatomy, physiology, and biochemistry are the three major subjects offered in the first year of a traditional medical curriculum. Biochemistry needs to be taught and learned effectively in the context of the disease or a medical problem to facilitate the transfer of this knowledge in terms of diagnosis and treatment of the patients once they graduate and practice in the community (1).

Saveetha Medical College and Hospital was established in 2008, with an aim to provide quality medical education by adopting and implementing the active and self – directed learning strategies in its curriculum. The first batch of medical students was admitted during the academic year 2008–2009, and like in other medical schools in India, biochemistry is taught through didactic lectures. Since the institution was determined to change the teaching from passive to active learning, during the academic year 2008–2009, a study was conducted among the first-year MBBS students to see the effect of integrated teaching with case-based learning (CBL) in biochemistry (1). It was evident from the results of this study that the CBL improved learning gains as compared to the traditional curriculum (1). It was also observed that the students were able to formulate the learning objectives, were able to take responsibility for their own learning, and were able to gain knowledge by virtue of case scenarios used in the CBL strategy (1). Despite the CBL, students were unable to organize and integrate information and relate and link the basics of clinical biochemistry to medical problems. This is the most important lacuna that we have identified, and we strongly felt the need for the introduction of another innovative tool in combination with the clinical cases to fill this gap.

In this context, we conducted the literature review to find a solution for the problem identified and found that a few studies in physiology education have emphasized the use of concept mapping as an important tool to facilitate active, self-directed, and deep learning (2, 3). Schmidt describes the conceptual framework that promotes deep learning in a study about the Foundations of PBL (1993), where he describes six principles of cognitive learning, one of which deals with ‘elaboration’ of a content area (4). In concept-mapping sessions with clinical cases, elaboration of the clinical cases promotes the understanding and better retention of a content area. During the past decade, the use of concept mapping in medical education provided a chance for the medical students to improve their meaningful and deep learning (5, 6). However, the relevance and the use of concept mapping in medical biochemistry education were not studied and this is the rationale for developing this innovative curriculum.

Specific objectives of this study are to: 1) design and implement the innovative curriculum consisting of concept mapping with clinical cases, 2) compare the knowledge of the undergraduate medical students in the traditional curriculum with that of the students in the innovative curriculum, and 3) know the perceptions of the undergraduate medical students on the usefulness of the concept mapping with clinical cases in learning biochemistry.

## Materials and methods

The clinical case units consisted of a series of three common medical problems and symptoms, each followed by a set of case-related questions. These clinical case units were prepared as described in the previous study (1) with few modifications (see Box 1). All current first-year MBBS students (*n*=150) admitted during the academic year 2011–2012 attending the biochemistry course were randomly divided into two groups: the ones in the traditional, lecture-based program (*n*=75), and the ones in the new innovative, clinical cases with concept-mapping program (*n*=75). This study was conducted from December, 2011, to May, 2012. Due permission was obtained from the Institutional Ethics Committee before the start of the study. The 75 students in the innovative group were further randomly divided into groups of five, for a total of 15 small groups. Three units for concept mapping with clinical cases were used for this study. The clinical cases were given to the students for discussion in the small groups, including the preparation of concept maps, all under the supervision of a faculty member. The following steps were adopted for concept mapping as described by Novak and Canas with little modifications: 1) brainstorming stage, 2) organizing stage, 3) layout stage, 4) linking stage, 5) revising and finalizing stage, and 6) gallery walk (7). During this study, the same faculty member taught both groups of students, and the students of both groups had the same amount of teaching and learning time. Care has been taken so that both groups have the opportunity to clarify understanding by asking for help from the faculty member. Also, the time lag between the teaching and learning sessions and the assessment for each group was similar.

Box 1. An example of a clinical case unit

Topic: Cardiovascular System – Coronary Artery Disease

A 50-year-old male executive from a corporate firm comes to the Saveetha University Medical College Hospital complaining of recent pain in his chest that went to his left shoulder. This episode occurred after a brisk 10-min walk. The executive explained that the pain disappeared after he rested. He is suspected of having angina pectoris.

Specific Learning Objectives

By the end of the session, the students should be able to:

1. Explain the mechanism of plaque formation in the arteries
2. Enumerate the risk factors in coronary artery disease
3. List the normal and abnormal blood parameters in coronary artery disease
4. Explain cholesterol metabolism
5. Learn and interpret the normal and abnormal values of cholesterol
6. Retrieve the names of the compounds/drugs that reduces the cholesterol, lipids in the body and explain their role in clinical medicine
7. List the bile acids and explain how bile acids can be synthesized in our body
8. Illustrate the primary sources and precursors of cholesterol in the body
9. Explain how dietary cholesterol can be restricted
10. Illustrate and implement the protocol for the diagnosis of myocardial infarction in laboratory
11. List the biochemical markers for an acute myocardial infarction
12. Name and classify lipoproteins
13. Appreciate the biochemical role of lipoproteins in coronary artery disease

Areas to develop proficiency related to the objectives:

1. Cholesterol and other sterols that are important in normal metabolism and in disease states
2. Biosynthesis, metabolism, and excretion of cholesterol and bile acids
3. Role of cholesterol in the development of atherosclerosis and the relationship of hypercholesterolemia and dietary fat intake in this disease
4. Cholesterol normal values and interpretation of abnormal values
5. Hypocholesterolemic and hypolipidemic drugs
6. Biochemical tests used in the diagnosis of myocardial infarction
7. Estimation of creatine kinase (CK) over a period of time
8. Significance of estimation of CK, lactate dehydrogenase (LDH), and transaminase in the diagnosis of myocardial infarction
9. Biochemical role of lipoproteins and functions
10. Importance of lipoprotein (a)
11. Importance of heparin

Self-evaluation:

1. Define coronary artery disease
2. Enumerate the common risk factors in coronary artery disease
3. Write the normal values of the following biochemical parameters in blood:
  - Cholesterol
  - triglycerides
  - LDL-cholesterol
  - HDL-cholesterol
4. Explain how Atherosclerosis can be prevented
5. Name the hypolipidemic drugs
6. Write the name of the key enzyme of the cholesterol biosynthesis pathway
7. Hypercholesterolemia is thought to be related to the development of atherosclerosis – justify this statement
8. Lowering of the plasma cholesterol concentration is potentially beneficial for this patient – explain.
9. Describe the mechanism involved in producing the atherosclerosis
10. How cholesterol biosynthesis is normally regulated?
11. What role does HMG CoA-reductase play in cholesterol biosynthesis?
12. Explain the purpose of prescribing a drug that inhibits HMG CoA-reductase
13. List the biochemical markers available for the diagnosis of acute myocardial infarction
14. What is the relative merit of estimation of CK, LDH, and transaminase in the diagnosis of myocardial infarction
15. What is the relation of CK activity in the blood to tissue damage?
16. Explain the role of myoglobin in the diagnosis of myocardial infarction
17. Analyze the advantage of estimation of troponins in the diagnosis of myocardial infarction
18. What is heparin and mention the importance of heparin in management of coronary artery disease

Student performance was assessed using the scores from the three post-module written tests. The test contained multiple choice questions (MCQs), structured essay questions (SEQs), and short answer questions (SAQs). On an average, each test contained eight questions, that is, five MCQs, one SEQ, and two SAQs; the maximum score for each test was 20.

Statistical analyses to compare groups 1 (traditional program) and 2 (innovative program) were done using Student t-tests (SPSS version 15 for Windows).

The students in the innovative program were asked to complete a 12-item, 5-point Likert scale questionnaire regarding their perception of the usefulness of the concept-mapping approach in combination with the clinical cases, which was prepared based on the directions documented by Pinto and Zeitz (8). The questionnaire assessed the following issues: deep understanding of the subject matter, relevance of the cases, opportunities for discussion, use of critical thinking, usefulness of concept mapping, relevance for future practice, promotion of self-assessment, promotion of active learning, motivation to learn, meaningfulness of learning, and the role of the teacher with an open-ended question at the end of the questionnaire.

## Results

On all three tests administered, the students in the innovative curriculum with the concept-mapping group (range: 12.33–13.93) significantly outperformed (all *p*-values <0.001) the ones in the traditional lecture-based group (range: 7.13–8.28); see Table 1 for details.

**Table 1:** The mean±SD values of the test scores to assess the academic performance in group 1 (controls) and group 2 (study subjects)

Test	Group1: traditional program (*n*=75)	Group2: innovative program (*n*=75)	Effect size(Cohen's *d)*
1	7.99±2.34	12.33±3.17***	−0.61
2	8.28±1.40	13.93±1.67***	−0.88
3	7.13±1.26	13.3±1.51***	−0.91

*** *p<*0.001 compared to controls.

Virtually all of the students felt that the clinical cases were interesting and thought that the concept maps helped them better understand the biochemical topics. Participants opined that by virtue of the concept-mapping technique they were able to integrate the information and relate and link the basic concepts in biochemistry in terms of the clinical problem. They valued the exchange of ideas that took place in the small group discussions of the clinical cases followed by the concept mapping in small groups, thus making biochemistry much more fun and meaningful. The students felt that the clinical cases and concept maps promoted meaningful learning and deeper understanding of biochemistry compared to the traditional classroom teaching. Our data are supported by the results of various other studies (9, 10). Also, the participants in the innovative curriculum felt that the teacher's role as facilitator is the key factor for the proper conducting of these concept-mapping sessions in small groups.

## Discussion and conclusion

This innovative program consisting of concept mapping in conjunction with clinical cases improved the learning environment of the students and resulted in better test scores in biochemistry for the first-year medical curriculum. Our results are supported by various other studies that show instructional strategy using concept mapping as an innovative tool, which provides opportunities for developing and practicing contextual thinking and clinical collaboration skills (2, 3, 11).

The use of concept mapping along with the clinical cases stimulated meaningful learning on the part of the students (8). The students indicated that concept mapping promoted meaningful discussion of the clinical cases in the brainstorming stage; stimulated deep analysis of the medical problem in the organizing stage; facilitated the learning process to organize and integrate information in the layout stage; linked, related the basic biochemical concepts to medical problem in the linking stage; provided an opportunity to assess existing knowledge, gain insights about new and existing knowledge in the revising and finalizing stage, and exchange information by going through all the concept maps made by fellow students during the gallery walk. These findings strongly confirm that concept mapping is a useful strategy to promote meaningful learning in medical education (3, 11).

To conclude, our study results showed that the use of concept-mapping techniques resulted in significantly better test scores and promoted a deeper understanding of the basic concepts of biochemistry to relate and link to medical problems.
